# Quantitative imaging of doxorubicin diffusion and cellular uptake in biomimetic gels with human liver tumor cells

**DOI:** 10.1007/s13346-023-01445-1

**Published:** 2023-10-12

**Authors:** Oliver Degerstedt, Paul O’Callaghan, Ada Lerma Clavero, Johan Gråsjö, Olle Eriksson, Erik Sjögren, Per Hansson, Femke Heindryckx, Johan Kreuger, Hans Lennernäs

**Affiliations:** 1 Department of Pharmaceutical Biosciences, Uppsala University, Uppsala, Sweden; 2 Department of Medical Cell Biology, Uppsala University, Uppsala, Sweden; 3 Science for Life Laboratory, Uppsala University, Uppsala, Sweden; 4 Department of Medicinal Chemistry, Uppsala University, Uppsala, Sweden

**Keywords:** Doxorubicin, Drug diffusion, Miniaturized chip, Cellular uptake, Drug delivery, Liver cancer

## Abstract

Novel tumor-on-a-chip approaches are increasingly used to investigate tumor progression and potential treatment options. To improve the effect of any cancer treatment it is important to have an in depth understanding of drug diffusion, penetration through the tumor extracellular matrix and cellular uptake. In this study, we have developed a miniaturized chip where drug diffusion and cellular uptake in different hydrogel environments can be quantified at high resolution using live imaging. Diffusion of doxorubicin was reduced in a biomimetic hydrogel mimicking tissue properties of cirrhotic liver and early stage hepatocellular carcinoma (373 ± 108 µm^2^/s) as compared to an agarose gel (501 ± 77 µm^2^/s, *p* = 0.019). The diffusion was further lowered to 256 ± 30 µm^2^/s (*p* = 0.028) by preparing the biomimetic gel in cell media instead of phosphate buffered saline. The addition of liver tumor cells (Huh7 or HepG2) to the gel, at two different densities, did not significantly influence drug diffusion. Clinically relevant and quantifiable doxorubicin concentration gradients (1–20 µM) were established in the chip within one hour. Intracellular increases in doxorubicin fluorescence correlated with decreasing fluorescence of the DNA-binding stain Hoechst 33342 and based on the quantified intracellular uptake of doxorubicin an apparent cell permeability (9.00 ± 0.74 × 10^–4^ µm/s for HepG2) was determined. Finally, the data derived from the in vitro model were applied to a spatio-temporal tissue concentration model to evaluate the potential clinical impact of a cirrhotic extracellular matrix on doxorubicin diffusion and tumor cell uptake.

**Graphical abstract:**

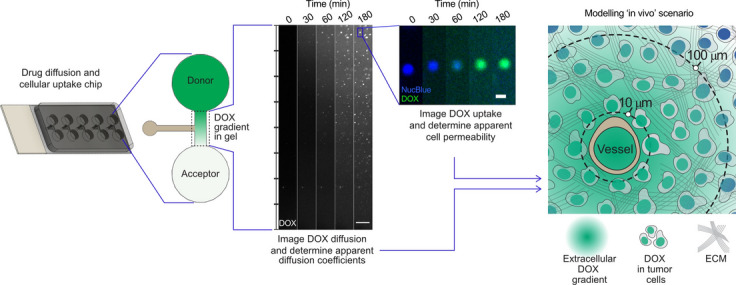

**Supplementary Information:**

The online version contains supplementary material available at 10.1007/s13346-023-01445-1.

## Introduction

In vitro 2D and 3D cell models form the backbone of pre-clinical cancer research. Combining such models with microfluidic solutions, which are typically compatible with high resolution imaging techniques, permit more complexity to be studied in vitro and enables tumor-on-a-chip approaches which may be more clinically relevant within drug development [1, 2]. In such devices gradients of nutrients, oxygen and cellular waste products can be formed as a result of cell metabolism, pressure differences, convective transport and diffusion similar to that seen in vivo [3–5]. However, challenges in terms of drug adsorption to materials as well as physiological relevance of the applied volumes, ratios and flows remain [1, 2].

Therefore, simpler miniaturized systems without fluidics may be applied to create in vitro models in which concentration gradients of drugs can be established in various disease-relevant matrices, thereby focusing on crucial in vivo processes such as drug diffusion, cellular uptake, tissue exposure and drug action [5–7]. These properties are important to establish, as it will assist the development of drug molecules that effectively diffuse across the tumor extracellular matrix (ECM) to reach intracellular targets and erase all cancer cells, which is crucial to prevent cancer resistance and recurrence [6, 8]. Additionally, such properties may also be implemented in various *in silico* modelling approaches in order to translate in vitro findings to a wider clinical context [2, 9, 10].

Hydrogels containing tumor cells are often used for modelling tumor tissue in vitro and low melting point (65.5 °C) agarose is especially useful for the development of such novel miniaturized systems as it provides ease of preparation in aqueous media and gels at room temperature. Investigating diffusion in such gels may provide an accurate estimation of unhindered drug diffusion, i.e. free diffusion in water [10–12]. This in turn means that the Stokes-Einstein equation can be applied to predict, with good approximation, diffusion coefficients in such agarose hydrogels.

Hepatocellular carcinoma (HCC) is a primary liver tumor that usually develops on a background of chronic liver disease and progresses from fibrosis to cirrhosis [13]. These changes are characterized by the extensive deposition of the ECM proteins collagen and fibrin, which change the biomechanical properties of the liver and increase its stiffness as the disease progresses. This progression can be mimicked in vitro by the development of biomimetic hydrogels containing collagen and fibrin [14]. The gels stiffness can be tuned to biophysically resemble both healthy and diseased mice liver tissue [15], and the diffusion coefficients of piroxicam and human lactoferrin, which encompass a molecular mass (M) range from 331 to 79 000 g/mol, are significantly reduced in the biomimetic gels compared with diffusion in agarose gels [10].

Doxorubicin (DOX; M = 543.5 g/mol) is a clinically relevant and commonly investigated anticancer drug with two different mechanisms of action, which both activate apoptotic pathways. Firstly, DOX can intercalate between DNA base pairs to prevent DNA replication. The second mechanism of action is the intracellular generation of reactive oxygen species [16–18]. Severe side-effects associated with DOX treatment have been reported, such as cardiotoxicity, bone marrow toxicity and intestinal mucositis [19–21]. Therefore, locoregional treatments with DOX such as emulsion-based transarterial chemoembolization (TACE) [22], or systemic treatments using nanoparticles such as liposomal formulations [23], are attractive in the clinical setting as these strategies offer targeted drug accumulation at the tumor site as well as reduced side-effects.

The molecular structure of DOX allows for relatively simple quantification of drug concentration via both absorbance- and fluorescence-based techniques [24]. In biological in vitro settings fluorescence-based techniques have proven more useful since many hydrogel and cell culture components contribute challenging background absorbance in the UV spectrum [10]. Quantifying DOX via fluorescence must however be performed with caution since the fluorescence emission spectra for DOX overlaps with the emission spectra for some commonly used stains in fluorescence microscopy (e.g. the cell death reporter propidium iodide). Additionally, the fluorescence signal of DOX can be both enhanced or quenched by lipids and other cellular components [25, 26]. One solution to this challenge, initially proposed by Hovorka et al*.* [27], is to study the signal from Hoechst stains (e.g. H33342) as an indirect readout for DOX concentration, as the Hoechst molecule competes with DOX for DNA binding. Therefore, a decrease in the observed Hoechst signal correlates with an increase in the nuclear concentration of DOX and permits indirect assessment of intracellular DOX concentrations.

The primary objective of this study was to develop a miniaturized chip for fluorescence-based visualization and quantification of DOX diffusion in biomimetic hydrogels that mimic tissue properties of cirrhotic liver and early stage HCC. Secondly, human liver tumor cells were added to the biomimetic gels and the influence of cells on DOX diffusion, as well as intracellular DOX uptake, was investigated. Finally, the implications of our in vitro findings were translated to a clinical scenario using spatio-temporal tissue concentration modelling.

## Materials and methods

### Materials

Fibrinogen (from bovine plasma), sodium hydroxide (NaOH), thrombin (from bovine plasma) and calcium chloride (CaCl_2_) were from Sigma-Aldrich (Germany). Aprotinin, NucBlue live cell stain ready probes reagent (Hoechst 33342), low melting point agarose (TopVision) and all cell culture reagents were from Thermo-Fischer Scientific (Sweden). Doxorubicin hydrochloride was from Toronto Research Chemicals (Canada). Collagen (type I, from rat-tail) was purchased from Ibidi (Sweden). Phosphate buffer saline (PBS), pH 7.4, was also from Sigma-Aldrich and prepared by dissolving tablets in MilliQ water yielding a mixture containing 0.01 M phosphate buffer, 0.0027 M potassium chloride and 0.137 M sodium chloride. All prepared PBS solutions were degassed using sonication (15 min) before further use. DOX stock solutions at a concentration of 100 mM were prepared by dissolving the powder in dimethylsulfoxide, before aliquoting to brown 1.5 mL vials and storage in a freezer at -20 °C until use. Sylgard 184 polydimethylsiloxane (PDMS) elastomer kit was purchased from Silmid (UK).

### Cell culture

Hep G2 (HB-8065™) cells were purchased from ATCC (USA) and Huh7 cells were a kind gift from Dilruba Ahmed, Karolinska Institute, Sweden. Both the HepG2 and Huh7 cell lines tested negative for mycoplasma. Cells were routinely cultured at 37 ˚C with 5% CO_2_ in high glucose DMEM GlutaMAX™ supplemented (Cat No. 10569010) or in high glucose DMEM, HEPES, no phenol red (Cat No. 21063029) for imaging experiments. Both media were supplemented with 10% fetal bovine serum (Cat No. 10270106) and 1% penicillin–streptomycin (Cat No. 15140122). Cell culture medium was changed every 2–3 days.

### Manufacturing of a combined drug diffusion and cellular uptake chip

**Fig. 1:**
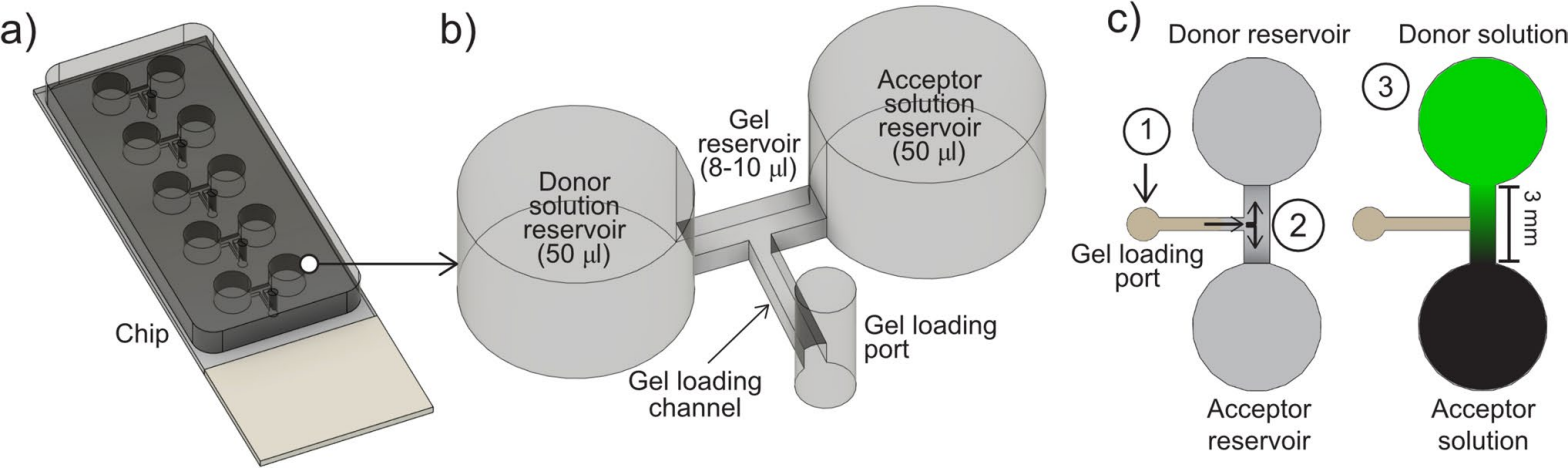
**a** The combined drug diffusion and cellular uptake chip and **b** zoom in on one of five gel reservoirs flanked by donor and acceptor reservoirs. **c **Top view of a drug diffusion and cellular uptake experiment where a gel (with or without cells) is loaded (1), once the gel has solidified the acceptor and donor solution reservoirs are filled (2), and finally the region of interest (ROI) is imaged as the drug concentration gradient (shown in green) is established (3).

The design for the miniaturized mold was drawn in Autodesk Fusion 360 (Autodesk, USA) and exported as a stereolithography (STL) file. The molds for polydimethylsiloxane (PDMS) casting were manufactured using a Form3 3D-printer (Formlabs, USA) and printed horizontally using black V4 resin (Formlabs, USA) and post-processed according to the manufacturer’s recommendations [28]. The post-processed molds were silanized overnight in a desiccator with 100 µl 1H, 1H, 2H, 2H-Perfluorooctyltrichlorosilane, 97% (ThermoFisher Scientific, USA). The (STL) file used for 3D printing is available upon request. The combined drug diffusion and cellular uptake chips were produced by first casting PDMS (Sylgard 184, 10:1 base:curing agent ratio) into the mold, degassing under vacuum for 2 h and then curing at 75 °C overnight. The PDMS device was then removed from the mold and holes corresponding to each gel loading port as well as two loading holes per reservoir were made using a 1.5 mm Miltex biopsy punch (Ted Pella Inc, USA). Finally, the PDMS was bonded to a glass slide (Superfrost, VWR) after plasma treatment using a BD-20AC laboratory corona treater (Electro-Technic Products, USA). To assure adequate bonding between PDMS and glass, the chip was sandwiched between aluminum plates and heated (75 °C) for 2 h. Each chip contained five replicates of one gel chamber (3 mm long × 1 mm wide × 0.6 mm high) connected to two adjacent solution reservoirs (donor and acceptor reservoirs) and a gel loading channel and port, see Fig. 1a, b for an illustration.

### Drug diffusion and cellular uptake experiments

The combined drug diffusion and cellular uptake chip (Fig. 1a–c) was developed to image and quantify DOX fluorescence in cell-laden hydrogels. The dimensions of the chip allowed for visual inspection of the gel-loading procedure without the need for magnification (Fig. 1c). The gel volume loaded (8–10 µL) was dependent on the hydrogel of interest. Once the gel solidified (typically after 15 min), first the acceptor solution (without DOX) and then the donor solution (containing DOX) were quickly pipetted (< 30 s) into the solution reservoirs (50 µL). The chip was placed under a confocal microscope (Supplementary Fig. 1b) for imaging of the region of interest (ROI) during the formation of DOX concentration gradients (Fig. 1c). The time elapsed between adding the drug solution to the start of image acquisition was noted for each replicate (*t*_lag_) and accounted for during subsequent diffusion determinations. Additionally, in gels-with-cells experiments, cell nucleus staining was performed with NucBlue (Hoechst 33342) to identify the number and distribution of cells. To avoid gradients of NucBlue staining solution forming, the stain was added to the cell media (12 drops/mL) used to prepare the gels. The diffusion and cellular uptake experiments were run for three hours.

### Preparation of hydrogels

Low melting point agarose (LMPA) gels (1% w/v) were prepared by first adding agarose powder in PBS. The mixtures were heated in a water bath at 60 °C for 15 min and regularly vortexed in order for the LMPA to become completely dissolved. The LMPA gel was then cooled to 37 °C and carefully pipetted (8 µL) to the chip (Fig. 1c) for diffusion experiments. After all five gel reservoirs had been loaded, the chip was left at room temperature for 15 min for gelation to occur.

Biomimetic gels consisting of type I-S fibrinogen (from bovine plasma, F8630-5G, Sigma-Aldrich, Germany) and type I collagen (Rat-tail type I, 50,201, Ibidi, Sweden) were manufactured similarly as described by Calitz et al. [14, 15]. Final concentrations of fibrin and collagen were 30 mg/mL and 2 mg/mL, respectively. First, a 70 mg/mL stock solution of type I-S fibrinogen was prepared by weighing up 350 mg of fibrinogen, which was added gradually, under gentle agitation to a mixture of sterile PBS (4 mL), aprotinin (1 mL, 1218.75 KIU/mL) and CaCl_2_ (150 µL, 20 mM). The solution was incubated for over 2 h at approximately 30 °C in a water bath until completely dissolved. Fibrinogen stock solutions were stored in the dark at 4 °C and used within 72 h. Following that, single cell suspensions (Huh7 or HepG2, 1 × 10^6^ (LD) or 2 × 10^6^ (HD) cells/ml) were prepared and resuspended in DMEM. Subsequently, type I collagen (400 µL, 5 mg/mL, rat-tail type I, 50,201, Ibidi, Lund, Sweden) was neutralized with NaOH (7–8 µL, 1 M) and mixed with the cell suspension (162 µL). Finally, fibrinogen stock solution was added (428.5 µL, 70 mg/mL) to the mixture and converted to fibrin by addition of thrombin (1.5 µL, 0.1 U/ µL). Thereafter, 10 µl of the cirrhotic gels containing tumor cells were loaded in each gel reservoir of the chip (Fig. 1c). Cirrhotic gels without cells were prepared similarly as described, adding an equal volume of DMEM lacking the cell suspension.

### Confocal fluorescence microscopy imaging

All imaging was performed on an LSM 700 confocal laser scanning microscope (Carl Zeiss, Germany) with a plan-apochromat 10 × objective (0.45 numerical aperture). The experiments were run using a temperature-controlled stage at 37 °C. Fluorophores were excited with 405 nm or 488 nm laser lines, and emitted fluorescence was detected for spectral bandwidths relevant for NucBlue (Hoechst 33342) and DOX, and collected as separate channels. Images were acquired with Zen 2011 SP7 software (Carl Zeiss, Germany) using a scan average of 2, with 16-bit pixel depths. Differential interference contrast (DIC) was detected through a transmitted light photomultiplier (PMT) channel. To capture the entire ROI tile scans (1 × 5 tiles) of each gel reservoir were collected at regular time intervals (imaging every 5 or 10 min).

### Image analysis

**Fig. 2:**
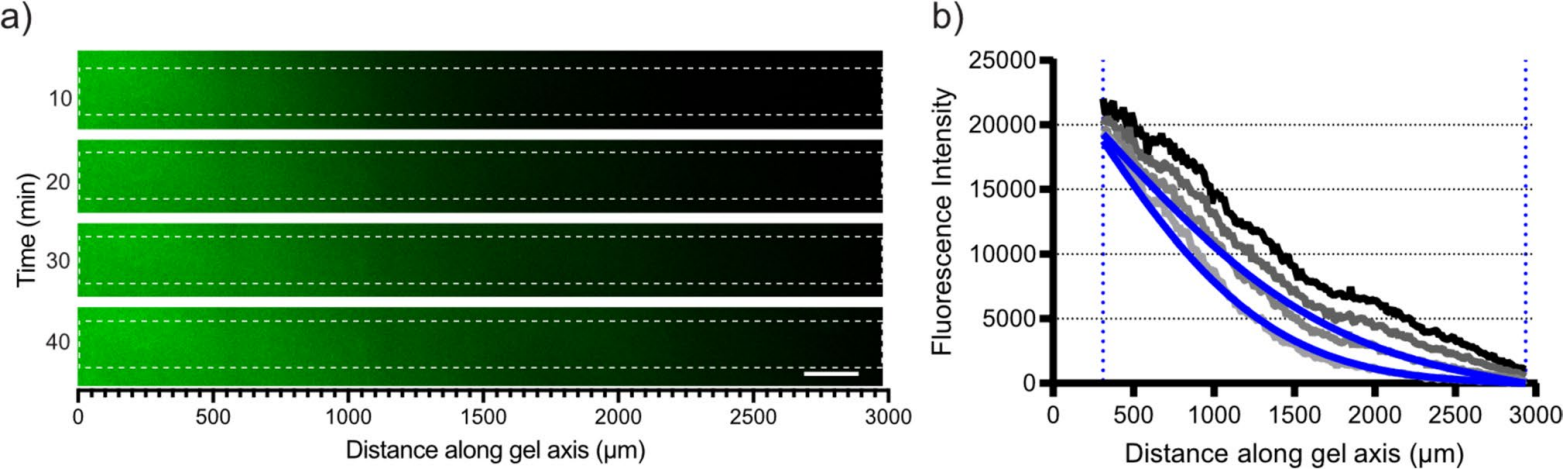
**a** Representative examples of DOX fluorescence gradients (here shown in green) formed in LMPA gel (PBS) reservoirs at 10, 20, 30 and 40 min (scale bar; 200 µm). White boxes indicate the selected ROI where DOX fluorescence intensity was recorded and visualized in profiles exemplified in **b**. The light gray curve corresponds to the 10 min profile and the black curve to the 40 min profile, blue curves show the fitted fluorescence profiles (see Materials and Methods for additional information) at 10 and 20 min in order to determine the apparent DOX diffusion coefficient. The blue dotted lines correspond to the chosen lower and upper boundary of the gels length

Images were collected as.czi files using the Zen 2011 SP7 software and opened in the Fiji version of ImageJ [29]. The NucBlue, DOX and DIC channels were split into separate time-series, and the DIC channel was used to identify the physical boundaries of the gel and crop all channels accordingly. For the determination of diffusion coefficients, a ROI was identified in the DOX fluorescence channel by using the straight line tool (width 100 µm) from the donor reservoir to the acceptor reservoir, straight through the entire length of the gel (3 mm, Fig. 2). The average fluorescence intensity across the x-direction for each y-coordinate (distance along gel axis, µm) was extracted using the “StackProfileData” macro and transferred to Microsoft Excel for continued analysis [30]. The fluorescence intensities were baseline (background) corrected with one of two approaches prior to DOX diffusion coefficient determinations. Either an average fluorescence intensity of the 100 µm segment of the gel furthest from the DOX donor solution, or the entire fluorescence intensity profile, at t = t_lag_ was used for baseline correction (see Supplementary Fig. 3 and Supplementary Table 1 for more details). In each experimental replicate, baseline corrected DOX fluorescence profiles at two different time points between 10 to 40 min were used to determine DOX diffusion coefficients (exemplified in Fig. 2).

For cellular uptake analysis, the NucBlue channel was initially used for each replicate to identify the number and distribution of cells in the gel (Fig. 4). This was done using Fiji to (i) prepare a maximum intensity projection of the entire time series, (ii) threshold the NucBlue signal to ensure detection of all nuclei in the channel, (iii) dilating the boundaries of the NucBlue threshold signals and if needed applying the watershed function to delineate clusters before finally (iv) analyzing particles. This analysis identified the y-position in the gel for each nucleus (ROI-list) as well as summarizing the number of detected nuclei and their size (projected area). The dilated NucBlue projected area served as a good approximation of the cell area (when compared with DIC images) and was subsequently used to calculate the cell radius of analyzed cells. Then, the average NucBlue intensity for each cell was extracted, using the multi-measure analysis function, for further analysis. The generated ROI list was also applied to the DOX channel to extract average intensities per cell. In order to combine the different replicates on each chip, cell intensities were grouped and averaged in 250 long µm zones based on their y-position, where zone 0–250 µm was closest to the donor solution and zone 1750–2000 µm was furthest from the donor solution.

### Determination of apparent diffusion coefficients

Assuming that the fluorescence intensity was proportional to the DOX concentration (< 20 µM, Supplementary Fig. 2b), the apparent diffusion coefficients were determined by fitting theoretically generated fluorescence profiles to the measured baseline corrected fluorescence profiles. In this study fluorescence profiles were generated by solving the diffusion equation numerically by the Crank–Nicholson algorithm [31], where the separation of the temporal points were 30 s and the separation of the spatial points were 12.50339 µm, corresponding to 10 pixels in the measurement setup. Using every 10th pixel measurement, substantially reduced the time for the curve fitting described below in this section, without compromising the quality of the fits. With this algorithm, fluorescence profiles for a specified diffusion coefficient were generated in a chosen interval of the gel. The upper boundary of that interval was set at the interface of gel to receiver solution with the condition that the concentration (fluorescence intensity) is zero at all-time points, which is consistent with the sink condition assumption of the receiver solution. The lower boundary of the interval was chosen to avoid any irregularities in the recorded fluorescence signal from the region around the interface between gel and the donor solution. The boundary condition for the lower boundary (${I}_{LB}(t)$) used in the numerical solution was constructed in the following way. For the first two time intervals between the start measurement and the two consecutive measurements, a function according to Eq. 1 was used as a (time dependent) boundary condition, where the parameters ${I}_{LB,\infty }$ and $\beta$ were obtained by fitting Eq. 1 to the fluorescence measured at the two first time points at the lower boundary implicitly assuming that the fluorescence is zero at t = 0. To reduce noise effects, the intensity values at the lower boundary used in the fit was calculated as the average of the measured intensity at the lower boundary y-value together with the measured intensity for the closest 5 lower and 5 higher y-values. Using Eq. 1 was done to catch the in general concave intensity vs time profile showing up in the time interval up to the second time point of measurement. In the two remaining time intervals, function determined from linear spline was used as lower boundary condition. In some cases the experiment showed the intensity vs time profile that was linear or slightly convex in the time interval up to the time point of the second measurement and in these cases the whole ${I}_{LB}(t)$ - profile function was approximated by linear spline in all time intervals. The time (*t*) used in Eq. 1 included the lag time (*t*_*lag*_) from addition of the donor solution to the chip to the start of imaging in the microscope.

1 $${I}_{LB}\left(t\right)={I}_{LB,\,\infty }\cdot \left(1-{e}^{-\beta \cdot t}\right)$$

The determination of the apparent diffusion coefficients was carried out by an in house developed Visual basic macro, fitting Crank–Nicholson generated fluorescence profiles to the measured fluorescence profiles using a Levenberg–Marquardt curvefit algoritm. Fitting was performed in an interval from the above defined lower and upper boundaries except for experiments where boundaries were adjusted to avoid fluorescence intensity regions regarded as experimental artefacts (exemplified in Supplementary Fig. 4). Out of four extracted time points, two were selected based on the requirement that minimal fluorescence intensity should be present at the end of the gel closest to the receiver solution. This resulted in the use of earlier time points in faster diffusion matrices (e.g. 10 and 20 min profiles in LMPA gels, Fig. 2b and Supplementary Fig. 4a). Additionally, a substantial enough fluorescence profile (> 1 mm) was desired for curve fitting, resulting in the use of later time points in slower diffusion matrices (e.g. 20 and 30 min profiles in cirrhotic gels, Supplementary Fig. 4b). The fit procedure was regarded as converged when the relative difference between the sum of squared error from two consecutive iterations was less than 0.000001.

### Determination of intracellular uptake and apparent cell permeability

The average cellular DOX fluorescence intensities for each zone were converted to DOX concentrations using calibration curves (Supplementary Fig. 2b), and plotted over time resulting in a linear relationship between concentration and time. By linear regression, the intracellular uptake of DOX (µM / min) was determined as the slope of the concentration vs time relation. The obtained intracellular uptake for each zone was in turn then plotted over the gel concentration for the corresponding zone (µM) at three timepoints (60, 120 and 180 min) resulting in a linear relationship between intracellular uptake and concentration. By linear regression, the overall transport rate constant of DOX into the cell (k_cell_, min^−1^) was determined as the slope of the intracellular uptake vs concentration relation. The apparent cell permeability of DOX (P_cell_, µm/s) was then determined using Eq. 2:

2 $${P}_{cell}=\frac{{k}_{cell} \times {V}_{cell}}{{\mathrm{A}}_{cell} \times 60}$$

where V_cell_ and A_cell_ are the volume and area of the cells respectively. Approximating the cells are spherical, the ratio V_cell_ / A_cell_ can be simplified to *r*/3 where *r* is the cell radius.

### Spatio-temporal tissue concentration modelling

In order to translate our in vitro findings to a clinical scenario a previously published spatio-temporal tissue concentration model (Fig. 6a) was applied [10]. The model was built in the PK-Sim modeling software (Open Systems Pharmacology Suite 8.0, [32]) and was originally based on a DOX model by Hanke et al*.* [33], which was further adapted by Kullenberg et al*.* [23] and Degerstedt et al*.* [10]. Briefly, intracellular and extracellular DOX tumor concentrations at 10 and 100 µm from the nearest blood vessel were simulated following administration of a clinical bolus dose of 50 mg/m^2^ and using the in vitro determined apparent DOX diffusion coefficient and apparent cell permeability. All other model parameters employed have been previously published together with the original spatio-temporal tissue concentration model file [10].

### Statistics

**Fig. 3:**
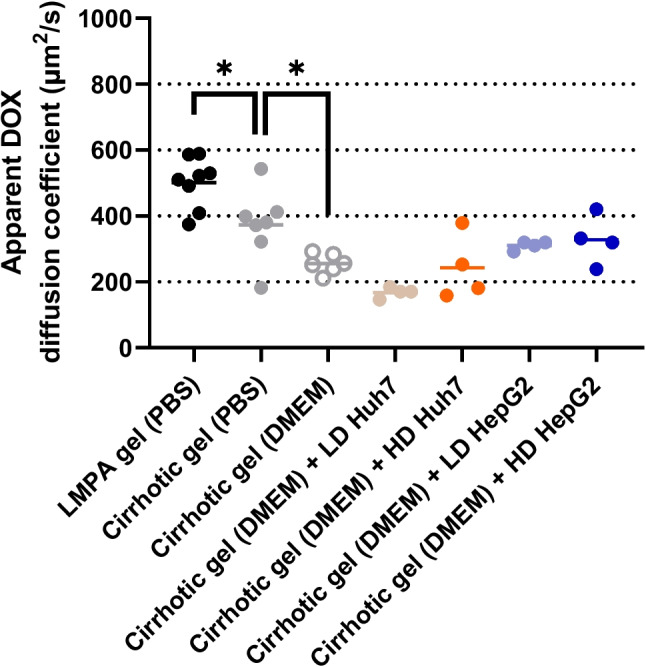
Scatterplot with mean values (horizontal line) for the determined apparent diffusion coefficients (µm^2^/s) of DOX in low-melting point agarose gels in PBS (LMPA, *n* = 8), cirrhotic gels containing no cells in PBS (*n* = 7) or no cells in DMEM cell media (*n* = 6) or in cirrhotic gels in DMEM cell media mixed with Huh7 or HepG2 cells (all *n* = 4). Low density (LD) and high density (HD) corresponds to one million and two million tumor cells per mL of cell media, respectively. An unpaired t-test for LMPA gel and Cirrhotic gel resulted in a *p* = 0.019 (*) and an unpaired t test with Welch's correction for Cirrhotic gel (PBS) and Cirrhotic gel (DMEM) resulted in a *p* = 0.028 (*)

In this study, the arithmetic mean is reported together with the standard deviations (SD) unless otherwise stated. The determined apparent diffusion coefficients for DOX (Fig. 3) in LMPA gel and cirrhotic gel were evaluated by a Shapiro-Wilks normality test and F-test to compare variances leading to the choice of an unpaired t-test to determine if the observed difference was significant. To evaluate the effect of medium choice in cirrhotic gels (PBS vs DMEM) the Shapiro-Wilks normality test passed but the F-test to compare variances pointed towards statistically different standard deviations (SD) and hence an unpaired t test with Welch's correction was used. To evaluate the addition of tumor cells to the cirrhotic gels the mean for each condition (with cells) was compared to the mean of the control condition (no cells in DMEM). The non-parametric Kruskal–Wallis with Dunn’s multiple comparisons post hoc test was used since the Shapiro-Wilks normality test did not pass. The increased CV% between LD or HD tumor cells in cirrhotic gels was evaluated using an F-test to compare variances for each cell line separately. Statistical significance was denoted by one star (*) for p ≤ 0.05. All statistical analysis was conducted using GraphPad Prism 9.0.0.

## Results

### DOX gradients were rapidly established in the gel reservoirs

The diffusion of DOX was visualized using fluorescence microscopy (Fig. 2a), and detectable as well as quantifiable fluorescence gradients were generally established across the entire gel reservoir (3 mm) within one hour (Fig. 2b). Linear calibration curves (R^2^ > 0.97) were established in the different gel matrices between 1 to 20 µM (Supplementary Fig. 2b). The apparent DOX diffusion coefficients were determined by curve fitting to the measured fluorescence profiles at two different time points (see Fig. 2b for an example and Materials and Methods for additional information). A selection of fits in different gel matrices are also presented in Supplementary Fig. 4.

### Biomimetic cirrhotic gels reduce DOX diffusion

Initially, the diffusion of DOX (20 µM) in low melting point agarose gels (LMPA, 1% w/v, pH = 7.41) and the biomimetic cirrhotic gel (pH = 6.86 ± 0.07), both prepared in PBS, were determined and compared. The apparent diffusion coefficient of DOX (Fig. 3) was significantly lower (*p* = 0.019) in the cirrhotic gel (373 ± 108 µm^2^/s) as compared to the LMPA gel (501 ± 77 µm^2^/s). Next, to support the subsequent addition of human liver tumor cells, the cirrhotic gels were prepared using DMEM cell media (pH = 8.61 ± 0.27), instead of PBS. This significantly lowered the apparent diffusion coefficient of DOX (Fig. 3) in cirrhotic gels from 373 ± 108 µm^2^/s to 256 ± 30 µm^2^/s (*p* = 0.028).

### Tumor cells did not influence DOX diffusion in cirrhotic gels

In an effort to model cirrhotic liver tissue and early stage HCC, biomimetic cirrhotic gels containing human liver tumor cells (Huh7 and HepG2) were prepared. The addition of a low density (LD) of Huh7 cells or LD HepG2 cells provided apparent DOX diffusion coefficients of 168 ± 16 and 310 ± 13 µm^2^/s, respectively. When a high density (HD) of Huh7 or HepG2 cells was employed the variability in DOX diffusion significantly increased, with CV increasing from 9 to 41% (*p* = 0.014) and 4% to 23% (*p* = 0.017), respectively. None of the observed effects of the added cells on DOX diffusion in the media were statistically significant when compared to the same gel matrix without cells.

### Liver tumor cells distribution throughout the gel reservoirs

**Fig. 4:**
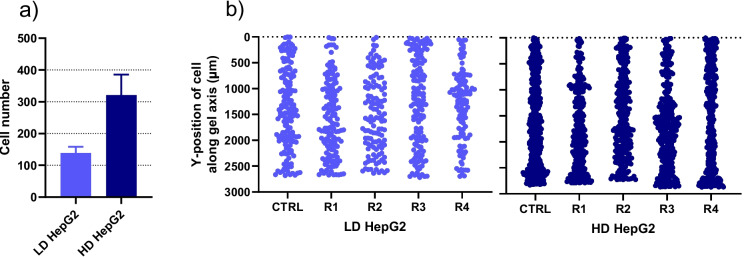
**a** The total recorded number of cells per gel reservoir (mean ± SD, *n* = 5) and **b **the distribution of all recorded cells (the y-positions in µm for each single cell along the gel axis is indicated) for the indicated control (CTRL) and experimental replicates (R1-R4) from chips with HepG2 tumor cells in cirrhotic gels. Low density (LD) and high density (HD) corresponds to 1 million and 2 million tumor cells per mL of cell media, respectively

**Fig. 5:**
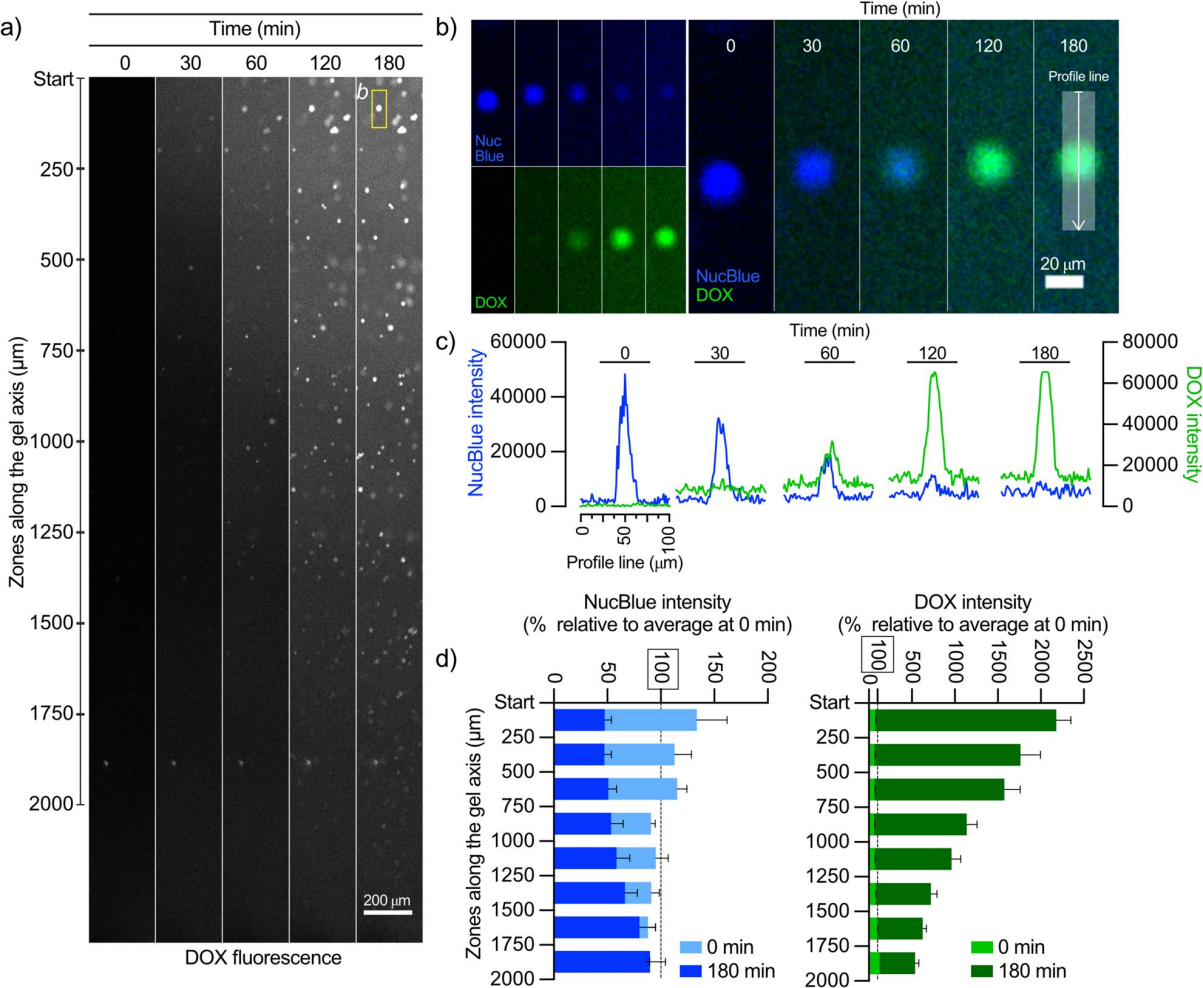
**a** Grayscale images from a representative drug diffusion and cellular uptake experiment highlighting the gel zones in the gel reservoirs and DOX fluorescence gradient formation over time. **b** DOX uptake and corresponding NucBlue reduction in representative cell images and **c **the corresponding quantifications of fluorescence intensities along a profile line. **d** Relative cellular fluorescence intensities (from n = 4 gels, mean ± SD) of NucBlue (blue) and DOX (green) in a high density of HepG2 tumor cells (2 million cells / mL of cell media) in cirrhotic gels at 0 and 180 min

To visualize the distribution of HepG2 cells in the gel reservoirs, the live nuclear fluorescent stain NucBlue was diluted in the DMEM cell media used to prepare the cirrhotic gels. Mixing 1 million or 2 million HepG2 cells per mL of cell media in the cirrhotic gels resulted in the detection of 139 ± 19 or 321 ± 65 tumor cell nuclei/gel reservoir, respectively (Fig. 4a). The HepG2 cell radius was determined to be approximately 9.9 ± 0.7 µm from the images in these experiments and was later employed in the spatio-temporal tissue concentration model. The image analysis also determined the unique y-position in the gel for each detected nuclei, which allowed us to visualize the cell distribution (Fig. 4b) and extract cellular fluorescence intensities over time (Fig. 5).

### Intracellular uptake of DOX and apparent cell permeability

In order to facilitate further analysis, the cell nuclei y-positions in the gels were grouped in zones of 250 µm (Fig. 5a). Single cell analysis in the gel zone closest to the DOX donor solution (0–250 µm) revealed a distinct reduction of NucBlue fluorescence as the DOX concentration gradient was established over time (Fig. 5b). A profile plot of fluorescence under the line, which included gel upstream and downstream of a cell of interest demonstrated a clear enrichment of DOX signal in the cell nuclei, but no detectable depletion of the DOX signal downstream of the cell, relative to the DOX signal upstream of the cell (Fig. 5c). When looking across the entire 0–250 µm zone the relative cellular intensities of NucBlue were reduced to approximately 50%, meanwhile the relative cellular intensities of DOX had increased by up to 2000% after 180 min for the cells in the zone closest to the donor solution (Fig. 5d).

The mean cellular DOX fluorescence intensities in each zone were converted to DOX concentrations using calibration curves (Supplementary Fig. 2b) and then plotted over time to determine intracellular uptake rate of DOX (µM / min) in each zone of the gel. In experiments with the high density of HepG2 cells, the uptake rate of DOX ranged from 0.22 µM/min in the 0 – 250 µm zone to 0.03 µM / min in the 1750 – 2000 µm zone. Expressing this relative to the available DOX concentration in the gel for each zone at three time points (60, 120 and 180 min) resulted in an overall transport rate constant for DOX (k_cell_) of 0.0163 ± 0.001 min^−1^ and consequently an apparent cell permeability (P_cell_) of 9.00 ± 0.74 × 10^–4^ µm/s. The lower density of HepG2 cells resulted in slightly higher maximum cellular uptake rate (0.29 µM/min in zone 0 – 250 µm), even if the k_cell_ and P_cell_ were lower, with 0.0120 ± 0.002 min^−1^ and 6.49 ± 1.25 × 10^–4^ µm/s, respectively.

### Translation of in vitro findings to a clinical scenario using modelling

**Fig. 6:**
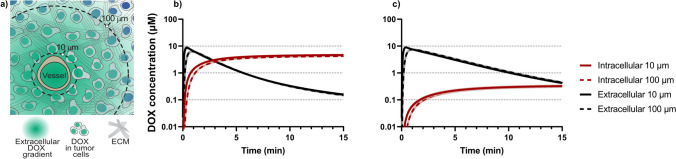
**a** Illustration of the employed spatio-temporal tissue concentration model used to simulate intracellular (red) and extracellular (black) DOX concentration–time curves **b**, **c** at 10 and 100 µm from the nearest blood vessel after an intravenous bolus dose of 50 mg/m^2^. The simulations were generated using apparent DOX diffusion coefficients corresponding to those determined for the cirrhotic gel with a high density of HepG2 cells. In **c** the apparent cell permeability of DOX determined for a high density of HepG2 cells in a cirrhotic gel was utilized

The in vitro findings were finally used as input parameters in our previously published spatio-temporal tissue concentration model [10] to demonstrate a valuable translation of experimental in vitro data to a clinical scenario (Fig. 6). Extracellular and intracellular tumor concentration–time profiles following a 50 mg/m^2^ DOX intravenous bolus injection, were simulated using the model (Fig. 6b–c). The distances of 10 and 100 µm were selected based on the fact that that most tumor cells are only a few cell diameters away from the nearest blood vessel but also have been shown to be 100 µm or further away from blood vessels (illustrated in Fig. 6a) [6, 34]. Initially, simulations using the apparent DOX diffusion coefficient determined for cirrhotic gels with a high density (two million cells/mL cell media) of HepG2 tumor cells was used (328 ± 74 µm^2^/s, Fig. 6b) together with the previously published in silico generated P_cell_ (220 × 10^–4^ µm/s) [10, 33]. This led to a negligible difference between the DOX concentration–time profiles at 10 and 100 µm into the tumor, highlighting the central role of the extracellular matrix in modulating DOX penetration deeper into the tumor. Next, the effect of cell permeability was evaluated by applying the P_cell_ value determined for a high density of HepG2 tumor cells (9.00 ± 0.74 × 10^–4^ µm/s, Fig. 6c). This reduced the observed tumor intracellular DOX concentrations by a factor ten (from approximately 5 to 0.5 µM).

## Discussion

In this study, an easy-to-use chip was cast from a reusable mold that was produced using 3D-printing. The chip enabled fluorescence-based visualization and quantification of doxorubicin (DOX) diffusion in biomimetic tumor cell-laden hydrogels formulated to model cirrhotic liver tissue and early stage HCC. Biomimetic hydrogels can exhibit extensive background absorbance when using UV-based imaging techniques, making the quantitative study of drug diffusion from solution to gel challenging [10, 35]. In addition, extended UV-light exposure may be detrimental for the drug and the hydrogels as well as any added human cells [10, 36]. By using a fluorescence-based technique and developing a miniaturized drug diffusion and cellular uptake chip, these limitations were mitigated. The relatively simple operation of the chip, avoiding the need for pumps, tubing, flow control and repeated pipetting was an advantage when working with a toxic, highly potent and material-adsorbing drug such as the cytotoxic DOX [37]. Notably, the chip may also be employed to investigate diffusion and gradient formation of other molecules with inherent or labelled fluorescence. It could therefore be applied to assess diffusion and uptake characteristics of other drugs designed for locoregional treatments of solid tumors in other organs, such as prostate, bladder or ovarian cancers [38–40]. Lower concentration LMPA gels (0.5% w/v) as well as gel preparations without fibrinogen were also evaluated in the chip, but displayed inadequate gelation properties and subsequently increased the risk of acceptor or donor solutions flushing through the gel reservoirs upon addition to the solution reservoirs (data not shown).

The lower apparent diffusion coefficient for DOX in cirrhotic gels (373 ± 108 µm^2^/s) compared to LMPA gels (501 ± 77 µm^2^/s) was in line with our previous findings (Fig. 3). Fibrin gels (30 mg/mL) and 1% LMPA gels have reported mesh sizes of approximately 50 nm and 600 nm, respectively, which suggests size-exclusion effects may contribute to the lower DOX diffusion in cirrhotic gels [41, 42]. However, for molecules below the mesh size, such as DOX monomers (diameter ≈1.5 nm [10]), electrostatic interactions have been suggested as the main determinant of diffusion in the ECM as well as in biological hydrogels [43–45]. At the physiological pH employed in these gels, pH = 7.41 in LMPA gels and 6.86 ± 0.07 in cirrhotic gels, DOX is expected to be positively charged (primary amine pKa between 8 and 9) [10, 46–48]. Even if the net charge of collagen gels at physiological pH was proposed to be zero [49], there may be pockets of negatively charged residues in the cirrhotic gels that can interact with the positive charge on DOX to slow down diffusion through the biomimetic ECM.

The larger magnitude (approximately factor 2) of the apparent DOX diffusion coefficients in this study compared to our previous results was somewhat surprising [10]. We considered a number of explanations for this apparent disparity, including that the temperature in the fluorescence microscope incubator (37 °C, Supplementary Fig. 1b) was higher than that in the UV-imaging instrument (ca. 30 °C) in which the previous experiments were performed. According to the Stokes–Einstein equation, the predicted free diffusion in water for a molecule should increase by 18% by increasing the temperature from 30 °C to 37 °C (from 362 µm^2^/s to 427 µm^2^/s for doxorubicin specifically). Additionally, studying diffusion from a gel *to* a solution [10] or to a gel *from* a solution (as described here) may play a part since DOX retention in the gel may be higher than in the aqueous solution, which would then reduce the diffusion. Finally, the lower DOX concentration employed (20 µM here in contrast to 1000 µM previously) may also impact diffusion. DOX is amphiphilic and self-associate trough one or more hydrogen bonds at around 1000 µM [50]. Therefore a lower drug concentration in solution should result in a larger fraction of monomeric DOX available to diffuse, and accordingly a higher observed diffusion rate. The 20 µM DOX donor concentration also provides more clinically reasonable drug concentration gradients (1–20 µM) in the chips gel reservoir. In a clinical context, the local (vena cava) and systemic C_max_ for DOX was reported to be 2.21 and 1.77 µM respectively, following a 50 mg dose of DOX in a lipiodol-based emulsion to HCC patients (via conventional TACE) [51].

The apparent diffusion coefficient of DOX was further lowered (Fig. 3) when the gel medium was changed from PBS (373 ± 108 µm^2^/s) to DMEM cell media (256 ± 30 µm^2^/s), which was necessary to support the tumor cells that were subsequently added. The cell media contained 10% fetal bovine serum, which contains albumin that binds DOX and subsequently leads to a lower free drug concentration available for diffusion. The plasma protein binding of DOX in vivo has been found to be in the range of 74–82% [52]. This was also in line with previous in vitro reports where the diffusion rate of DOX was reduced by 17% when increasing the amounts of fetal bovine serum (from 5 to 50%) in cell media [53]. Additionally, using DMEM cell media instead of PBS to prepare the cirrhotic gels resulted in a pH increase from 6.86 ± 0.07 to 8.61 ± 0.27 which will result in a lower degree of charged DOX monomers in these gels. In this study we observed a clear trend towards a reduction of the apparent DOX diffusion coefficients as the total protein concentration in the gels increases, which is also in line with a previously described interaction filtering mechanism [43, 45]. Interestingly, the addition of two different types of liver tumor cells (Huh7 and HepG2), at two different densities, to the cirrhotic gels had no significant effect on the determined apparent diffusion coefficients (Fig. 3). Tortuosity is a concept used to describe the increased effective path length that molecules diffusing through an extracellular matrix between tumor cells will encounter [54, 55]. Ramanujan et al. used tortuosity to explain the observed difference between their obtained diffusion coefficients in collagen gels compared to those measured in mouse xenograft tumors [54]. Additionally, the fraction of a tumor accounted for by the extracellular space has been reported to range from 20 to 60% [56, 57]. In this study, the fraction of extracellular space in the gels was > 90%, which led us to conclude that this relatively low density of tumor cells present in the gel could not influence the diffusion of DOX. The spatio-temporal tissue concentration model (Fig. 6a) employed a value of 40% for the extracellular space fraction of the tumor. Based on the in vitro determined apparent DOX diffusion coefficients, the performed simulations suggested that negligible concentration differences would be observed at 10 and 100 µm from the nearest blood vessel, both in the tumors intracellular and extracellular locations (Fig. 6b). Although few reports in humans exist, Lankelma et al*.* observed a dramatic reduction in the DOX signal in the first 100 µm from the nearest blood vessel, when analyzing biopsies from breast cancer patients 2 h after IV dosing [58]. Studies in rodents are more common, where for example Patel et al*.* reported a reduced DOX signal to 40% at 100 µm from the nearest blood vessel (set at 100%) only 10 min after IV dosing in tumor bearing mice [59]. Based on the simulations in this study the DOX diffusion would need to be reduced by an additional factor of 25 to create a more distinct concentration gradient across the tumor tissue consistent with these in vivo observations.

In all diffusion and cellular uptake experiments, a quantifiable DOX concentration gradient (1-20 µM) across the gel was established within one hour. This allowed us to examine the effect of the concentration gradient on the human liver tumor cells for the remaining experimental time (in total three hours). The concentration gradient resulted in higher relative cellular DOX intensities for the cells located closer to the donor reservoir (> 2000%). During the same time, the NucBlue cellular intensities decreased by 50% in the same zone (Fig. 5d). This was in line with a previous report by Hovorka et al*.* where the decreasing H33342 signal was used to indirectly determine the intracellular uptake of DOX [27]. Similarly, Matsuba et al*.* studied the association of MS-247 (then a novel anticancer agent) with the minor groove of DNA by measuring the reduction in fluorescence intensity of H33342 in both cell-free and cellular assays [60]. Our findings therefore suggest that DOX is rapidly internalized in the cells, where it competes with NucBlue for DNA binding, and likely displaces it. NucBlue, which is a type of Hoechst dye, has a reported binding affinity to DNA of 1 – 10 nM and a molecular mass (M) of 452.6 g/mol [61], while the reported binding affinity to DNA for DOX (M = 543.5 g/mol) is in the range of 200 – 2000 nM [62, 63].

To assess this further we determined intracellular uptake rates (µM / min) based on the average cellular DOX concentrations. As expected the intracellular uptake rate was higher for cells closer to the donor solution (0.22 µM / min), e.g. in gel zones with higher extracellular drug concentrations. There was a slight effect of cell density such that a lower density of HepG2 cells resulted in higher maximum uptake (0.29 µM / min). This is in line with studies by Kobayashi et al*.* where the effect of reducing cytotoxicity by increasing tumor cell density (called the inoculum effect) was investigated in detail [64]. There, 10^6^ MOLT-3 tumor cells exhibited higher cellular content of DOX (pmol/10^6^ cells) than 10^8^ cells after a one hour exposure to approximately 2 µM of DOX. Here, the single cell analysis (Fig. 5b, c) suggests that while clearly internalizing DOX, its fluorescence immediately upstream and downstream of a cell are similar, indicating that the cells do not detectably deplete it from their surroundings. This further strengthens the theory that a substantially increased cell density would be required to further study any inoculum or tortuosity effects in this assay.

The determined apparent cell permeability of DOX in HepG2 cells was similar between experiments with low and high cell densities, 6.49 ± 1.25 × 10^–4^ µm/s compared to 9.00 ± 0.74 × 10^–4^ µm/s. In comparison, the cell permeability of DOX from a 10 µM solution flowing through a cylindrical microvessel coated with a MDCK cell monolayer was reported to be 32 ± 23 × 10^–4^ µm/s [65]. Since the apparent cell permeability determined here relies on DOX fluorescence signals in the tumor cell nuclei there are some important limitations worth scrutinizing. Firstly, we assume that the fluorescence signals completely originate from DOX and not potential degradation products that have been reported previously [25, 27]. Next, in this study, the overall intracellular DOX fluorescence signal was clearly enhanced over time (Fig. 5). This distinct intracellular uptake of DOX has been observed for both Huh7 and HepG2 cells previously in a 2D cell model, but then quantified by LC–MS [23]. However, binding to DNA is expected to quench the DOX fluorescence signal [25, 66], therefore the overall cellular signal enhancement observed in this study is most likely due to a combination of nucleus specific accumulation of the DOX monomer as well as unintended DOX binding to other cellular components. Similar signal magnification was observed by Chen et al*.* where the DOX fluorescence increased in the nuclei of HeLa cells by a factor of 20 over approximately 2 h when using fluorescence lifetime imaging [67]. The molecular self-quenching effects observed in this study are also consistent with the literature, as we observe a deviation from linearity between fluorescence and DOX concentration at around 25 µM (Supplementary Fig. 2a), a level, which was reached by the tumor cells closest to the donor solution within 90 min. This all suggests that we are more likely to underestimate rather than overestimate the determined DOX apparent cell permeability. When simulating DOX tumor concentrations using the in vitro determined apparent cell permeability a sharp decrease (from approximately 5 to 0.5 µM) of intracellular concentration was observed (Fig. 6b, c). In addition, we aimed to investigate the viability of liver tumor cells resulting from exposure to a DOX concentration gradient over time, to determine a cut off value for a “lethal concentration” for each cell type. However, as DOX fluorescence unfortunately interfered with both the conventional staining propidium iodide as well as the more far-red staining NucRed Dead 647, it made cell death studies using these stains technically impossible.

In conclusion, the combined diffusion and cellular uptake chip model developed here allowed for formation of a clinically relevant and quantifiable DOX concentration gradient (1-20 µM) within one hour in biomimetic gels with or without tumor cells. The early parts of each experiment, when the concentration gradient was under establishment, were used to study the drug diffusion process meanwhile the latter parts allowed for investigations into the intracellular uptake rate of DOX. Future applications for this model will be to evaluate other (less fluorescent) anticancer drugs coupled with live/dead and early apoptosis marker staining as well as study different biomimetic gels with higher tumor cell densities. Additionally, theoretical models will be established to better understand and translate the contribution of tissue drug diffusion and resistance mechanisms to the rate and extent of drug response in tumor tissues as well as in other complex and dynamic diseases. The combination of tumor cell uptake and in silico modelling will be an important tool to develop dose planning strategies for locoregional therapies, such as TACE in HCC, that aim to avoid strategies resulting in tumor tissue areas with sub-therapeutic drug exposure. For instance, such in vitro and in silico models are planned to be used in an on-going phase II study in patients with HCC as it is expected to improve the understanding of interactions between local pharmacology, tumor targeting, HCC pathophysiology, hypoxia, metabolomics and molecular mechanisms of drug resistance [68].

### Supplementary Information

Below is the link to the electronic supplementary material.

Supplementary file1 (DOCX 2241 KB)

## Data Availability

The datasets generated and analyzed during the current study are available from the corresponding author on reasonable request.

## Abbreviations

DOX: Doxorubicin
HCC: Hepatocellular carcinoma
ECM: Extracellular matrix
TACE: Transarterial chemoembolization
UV: Ultraviolet light
PBS: Phosphate buffered saline
LMPA: Low melting point agarose
SD: Standard deviation
ROI: Region of interest
DIC: Differential interference contrast
PMT: Photomultiplier
LD: Low density
HD: High density
