# Supplementary material for: Quantitative imaging of doxorubicin diffusion and cellular uptake in biomimetic gels with human liver tumor cells
Source: Drug Deliv Transl Res. 2023 Oct 12;14(4):970–83. doi: 10.1007/s13346-023-01445-1 (PMC10927899; doi:10.1007/s13346-023-01445-1)
Supplement: Supplementary file 1 — Supplementary file1 (DOCX 2241 KB) [file 13346_2023_1445_MOESM1_ESM.docx]

Quantitative imaging of doxorubicin diffusion and cellular uptake in biomimetic gels with human liver tumor cells

Oliver Degerstedt^1^, Paul O’Callaghan^2,3^, Ada Lerma Clavero^2^, Johan Gråsjö^1, 4^, Olle Eriksson^2,3^, Erik Sjögren^1^, Per Hansson^4^, Femke Heindryckx^2^, Johan Kreuger^2,3^ and Hans Lennernäs^1*^

1Department of Pharmaceutical Biosciences, 2Department of Medical Cell Biology, 3Science for Life Laboratory and 4Department of Medicinal Chemistry, all at Uppsala University, Uppsala, Sweden

*Corresponding author ([hans.lennernas@uu.se](mailto:hans.lennernas@uu.se))

## Supplementary Information

###
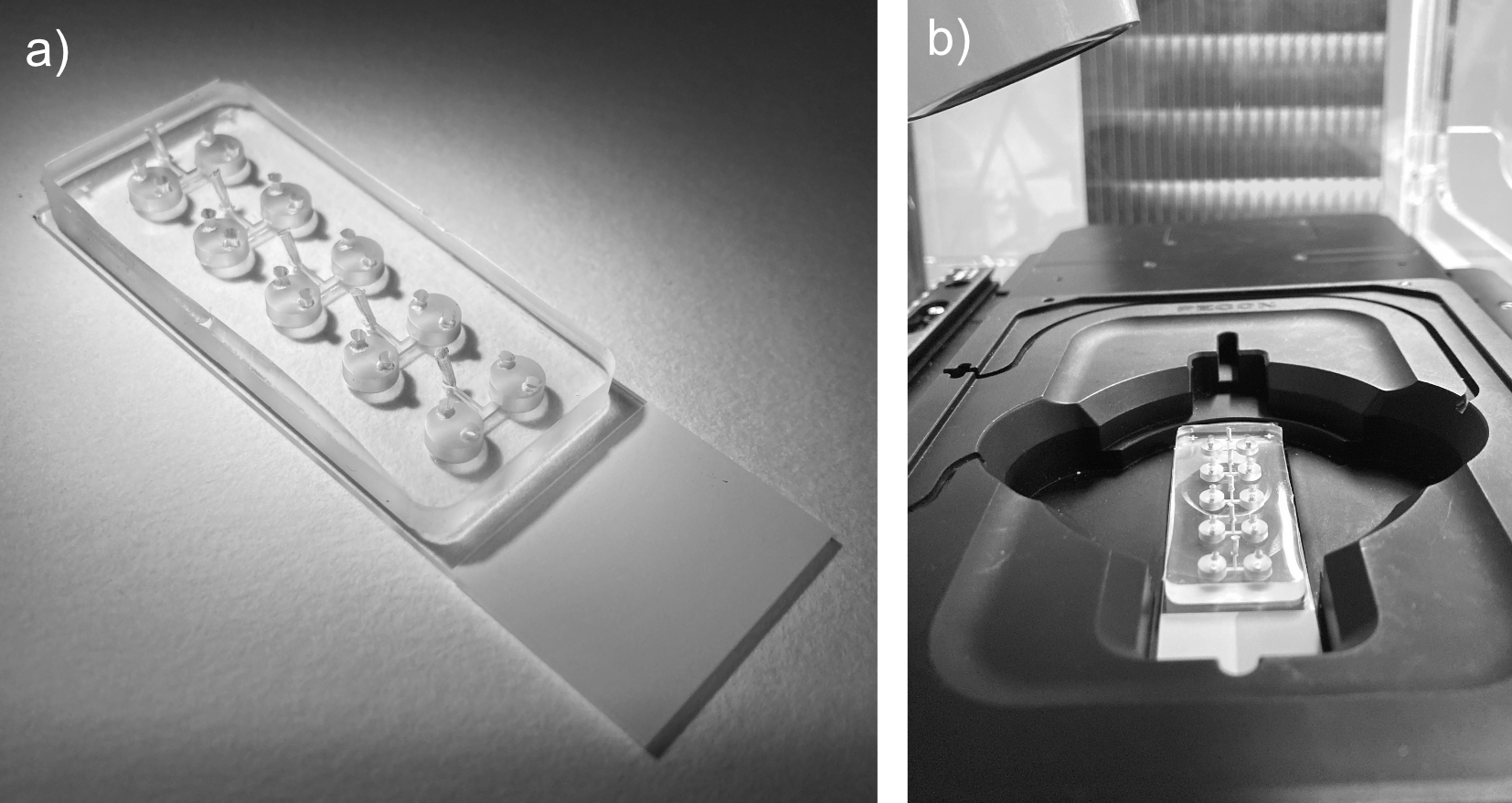
Images of chip and experimental set-up

**Supplementary Figure 1**. The drug diffusion and cellular uptake chip, a) close up image and b) mounted on the temperature controlled confocal microscope stage.

### Doxorubicin self-quenching and calibration curves

Deviation from linearity between fluorescence intensity and DOX concentration was observed above 25 µM (Supplementary figure 2a). Calibration curves for doxorubicin in different matrices (PBS, cell media, and cirrhotic gel) were constructed in the drug diffusion and cellular uptake chip (1 concentration / gel reservoir). The fluorescence intensities measured at the highest concentration (20 µM) were used to set the gain for the subsequent diffusion and cellular uptake studies.

**Supplementary Figure 2.** a) Non-linear calibration curve illustrating the deviation from linearity at DOX concentrations of 25 µM. b) Linearity of fluorescence intensity over doxorubicin concentration (1 – 20 µM in PBS and 0.8 – 20.5 µM in cell media). Excitation was with the 488 nm laser and emission was monitored between 535-700 nm in PBS and 535-600 nm in cell media. The shorter emission range in cell media was used in order to minimize bleed-through from the other staining to the DOX channel.

### Baseline correction of measured fluorescence intensities

Measured fluorescence intensities were baseline (background) corrected with one of two approaches prior to apparent DOX diffusion coefficient determinations.

In the majority of gel matrices (e.g. LMPA gel in PBS, black curve in Supplementary Figure 3) DOX had started diffusing into the gel reservoir by the time the first image was taken (at t = t_lag_). Therefore an average fluorescence intensity of the 100 µm segment of the gel (purple zone in Supplementary Figure 3) furthest from the DOX donor solution was used for baseline correction. These experiments are denoted with superscript “a” in Supplementary Table 1.

In experiments with cirrhotic gels containing a high density (HD) of HepG2 cells (blue curve in Supplementary Figure 3) the fluorescence intensities close to the DOX donor solution were negligible (< 1000) while the intensities closer to the receiver solution were not. Here, the entire fluorescence intensity profile at t = t_lag_ was used for baseline correction. These experiments are denoted with superscript “b” in Supplementary Table 1.

**Supplementary Figure 3.** Mean fluorescence intensity profiles (with shading as ± SD) of DOX in LMPA gel PBS (black curve) and Cirrhotic gel DMEM + HD HepG2 (blue curve) along the distance of the gel axis at t = t_lag_. The purple zone highlights the 100 µm segment of the gel furthest from the DOX donor solution used for baseline correction in most experiments.

### Fit of generated profiles to measured profiles

In general, measured baseline corrected fluorescence intensities covering as much as possible of the gel were used for fitting (Supplementary Figure 4c), however there were some exceptions where the lower (Supplementary Figure 4a and 4b) or upper (Supplementary Figure 4d) boundary of the fit were adjusted to account for experimental artefacts in the measured fluorescence profiles not accounted for by baseline correction.

**Supplementary Figure 4.** A selection of fits (blue curves) to measured baseline corrected DOX fluorescence profiles (grey-black curves), a) LMPA gel in PBS, b) cirrhotic gel in PBS, c) cirrhotic gel in DMEM cell media with a high density of Huh7 cells and d) cirrhotic gel in DMEM cell media with a high density of HepG2 cells. In a) and b) the 10, 20 , 30 and 40 min profiles are displayed, while the 30, 40, 50 and 60 min profiles are displayed in c) and d). Note different scale on y-axis (Fluorescence intensity).

### Checking the accuracy of the numerical solution routine

The accuracy of the fluorescence profile obtained from the numerical solution with the choice of temporal and spatial steps (i.e. Δ*t* = 30 s and Δ*y* =12.50339 µm) was checked by applying an algorithm appropriate for solving a similar diffusion problem in a simpler but slightly different system. The latter problem also has an analytical solution in the form of a series expansion as expressed by equation S.1 from J. Crank in 1975, Chapter 4, section 4.3.3, equation 4.22 [31], to which the numerical solution could be compared.

For this purpose we use a system where the upper boundary condition is a constant fluorescence intensity but all the other boundary and initial conditions are equal to the corresponding experimental conditions, and use typical values of the diffusion coefficient (400 µm^2^/s), gel length (3000 µm) and fluorescent intensity.

$$I\left( y,t \right)=I_{0}-I_{0}\frac{y}{l}-\frac{2\cdot I_{0}}{\pi}\sum_{n=1}^{\infty} \frac{1}{n}sin\left( \frac{n\pi y}{l} \right)\cdot e^{-n^{2}\cdot\pi^{2}\cdot Dt/l^{2}} (S.1)$$

Where *I* is the fluorescence intensity, *I*_0_ the fluorescence intensity of the donor solution, *y* the position in the gel counted from the gel interface to the donor solution, *t* the time and *l* the gel length. To generate the comparison between analytical and numerical solutions in Supplementary figure 5 we used the 20 first terms in the sum in eq. S. 1.

**Supplementary figure 5.** Comparison between numerical and analytical solutions for D = 400 µm^2^/s at t =300 s and 1500 s.

### Overview of determined apparent DOX diffusion coefficients in this study

Apparent DOX diffusion coefficients (µm^2^/s) were determined in three different ways during the progress of this study (Supplementary Table 1). First an analytical solution based on the DOX concentration profiles in the gel were employed, next a numerical solution based on the DOX concentration profiles was evaluated. Finally, the numerical solution based on the DOX fluorescence profiles was chosen as the optimal method since it allowed the best fit of generated profiles to measured profiles.

**Supplementary table 1.** Overview of determined apparent DOX diffusion coefficients in this study.
